# Medico-Chirurgical Transactions

**Published:** 1829-10-01

**Authors:** 


### VIII.

### CONTRIBUTION TO THE PATHOLOGY OF PHLEGMATIA DOLENS. By *Robert Lee,* M. D., &c. &c.

SUCH is the modest title of an interesting paper by Dr. Lee in the last volume of the Medico-Chirurgical Transactions. As Dr. Lee observes, there is perhaps no subject on which a greater difference of opinion now prevails than "on the pathology of phlegmatia dolens." By the way, the term pathology is strangely misapplied, for we see it continually used to express the anatomical characters of the disease, which is certainly incorrect. Morbid anatomy is a much more expressive as well as more precise expression. Dr. Lee goes on to remark that, the papers of Mons. Bouillaud, Dr. Davis, and Mons. Velpeau, first threw any light on the *real nature* of the disease, although he allows that they have not been sufficient to establish the *venous* origin of phlegmatia dolens. Since the Essay of Dr. Davis, published in the XIIth volume of the Medico-Chirurgical Transactions, the cases recorded in this country in support of the views of that gentleman, have been singularly unsatisfactory, and this consideration has led Dr. Lee to bring forward those which we now propose to notice.

*Case* 1. Mrs. J———, æt. 31, was delivered of her fifth child on the 10th of March, 1827, after a protracted labour, accompanied with severe pain shooting into her left thigh and leg. After the labour, this pain entirely subsided, and all went on well till the 14th. She then began to suffer from a sense of pain in the left groin and calf of the leg, with numbness in the whole left inferior extremity, and a slight tumefaction in the situation of the inguinal glands, where pressure occasioned great uneasiness. Occasional rigors—tongue furred—much thirst—bowels open—pulse 80—flow of milk and lochia natural. On the 16th, the pain was more severe, particularly from the groin to the knee on the inside of the thigh, where a swelling of a glistening white appearance was observed. On the 19th, the pain had diminished, but the swelling had greatly increased and extended to the leg and foot, which were both very tense and did not pit on pressure. The skin was not discoloured—the pain was relieved by moderate flexion of the limb. On the 24th, the pain was greater, the pulse quicker, the skin hot and moist, the patient extremely irritable and desponding. On the 25th, Dr. L. saw the patient for the first time.

"The whole extremity was much swollen, the intumescence being greatest in the ham and calf of the leg. The integuments wore a uniform smooth shining appearance, having a cream-like colour, and everywhere pitting on pressure, but more readily in some situations than in others. The temperature to the touch did not differ from that of the other limb, though she complained of a disagreeable sensation of heat throughout its whole extent, and much pain was experienced in the upper and inner part of the thigh on moving it. Immediately below Poupart's ligament, in the situation of the femoral vein, a thick, hard cord, about the size of the little finger was distinctly felt. This cord, which rolled under the fingers, and was exquisitely sensible, could be distinctly traced three or four inches down the thigh in the course of the femoral vessels, and great pain was experienced on pressure, as low down as the middle of the thigh in the same direction. The pulsations of the femoral artery were felt in the usual situation below Poupart's ligament; pressure over this vessel excited little or no uneasiness. Pulse ninety and sharp. Tongue much furred.—Thirst urgent. Bowels confined.—The lochial discharge had nearly disappeared." 135.

Leeches, cold lotions, cathartics, and anodynes were the means employed, and with success. For two months, however, the limb remained so feeble, as to disable her from walking, and continued larger than the other. Eleven months after the attack, her general health was restored and she again became pregnant, but on the 5th of November, 1828, after having given birth to a still-born child, she sank from uterine hæmorrhage.

*Dissection.* "The whole of the left inferior extremity was considerably larger than the right, but no serous fluid escaped from the incisions made through the integuments, beneath which a thick layer of peculiarly dense, granular, adipose matter was observed. The common, external iliac and femoral veins and arteries, enclosed in their sheath, were removed from the body for examination. The common iliac with its subdivisions, and the upper part of the femoral veins so resembled a ligamentous cord, that, on opening the sheath, the vessel was not, until dissected out, distinguishable from the cellular substance surrounding it. On laying open the middle portion of the vein, a firm thin layer of ash-coloured lymph was found in some places adhering close to and uniting its sides, and in others clogging it up, but not distending it. On tracing upwards the obliterated vein, that portion which lies above Poupart's ligament was observed to become gradually smaller, so that, in the situation of the common iliac, it was lost in the surrounding cellular membrane, and no traces of its entrance into the vena cava were discernible. The vena cava itself was in its natural state. The entrance of the internal iliac was completely closed, and in the small portion of it which I had an opportunity of examining, the inner surface was coated by an adventitious membrane. The lower end of the removed vein was permeable, but it coats were much more dense than natural, and the inner coat was lined with a strong membrane, which diminished considerably its calibre, and here and there fine bands of the same substance ran from one side of the vessel to the other. The outer coat had formed strong adhesions with the artery and the common sheath. The inguinal glands adhered firmly to the veins, but were otherwise in a healthy condition." 137.

The foregoing appearances were evidently those of inflammation at a remote period of time, and not of recent disease. A lithographic sketch is subjoined which shows the condition of the vein.

*Case* 2. Mrs. P———, æt. 27, of a delicate constitution, had been affected, during the last two months of pregnancy, with œdema of the right inferior extremity, a varicose state of the veins, and pain in the inside of the thigh and groin, especially severe at times in the latter. On the 8th of Dec. 1827, she was delivered, after a tedious labour, of a healthy child, and during the succeeding week felt unusual pain in the hypogastrium, and right iliac and inguinal regions. The pain then remitted but returned again on the 20th with augmented violence, and was followed on the 21st by rigors, pyrexia, and stiffness with swelling of the whole right inferior extremity. On the 22nd, Dr. Lee first saw her, (the patient belonged to Mr. Grant) when the extremity, more especially the leg, was much swollen, and the integuments of that part were tense and elastic, but pitted slightly on firm pressure along the front of the tibia. This was not the case in the thigh, but exquisite pain, aggravated by pressure, existed in the course of the femoral vein, which felt in the groin as if enlarged, rolling, hard, and incompressible beneath the finger. The pain extended upwards and downwards in the line of the great vessels—the temperature of the limb was increased—the pulse 112—the skin hot—tongue white—nausea—much thirst. On the 25th, all the symptoms were increased—the right labium pudendi was much swollen—and the integuments of the limb were pale, shining, and pitting every where on strong pressure. Next day a similar affection was observed to be commencing in the left extremity, and on the 27th there were fulness and severe pain in the left iliac region, and the swelling of the leg and temperature were increased. No enlargement of the femoral vein could be felt, but yet there was exquisite pain in its course through the groin and upper part of the thigh. On the 28th, the swelling of the right extremity having diminished, the indurated vein could again be felt in the groin, though the pain there was not much abated.

"The left extremity is swollen, stiff, hot, and painful, and cannot be moved, its integuments are white and pallid, and pit on strong pressure. The left labium pudendi is similarly affected. The femoral vein is felt enlarged and indurated in its passage through the groin;—intense pain marks its course down the thigh to the point where it leaves the ham; in this latter situation pressure produces much suffering, as it does down the whole posterior part of the leg. The parts of the limb out of the course of this vessel and of the great superficial veins are comparatively much less painful than on the lower and anterior part of the thigh; for several inches above the knee firm and continued pressure is borne without complaint. The countenance is pale and sunk. The pulse rapid. There is great irritability and prostration of strength." 141.

On the 29th, the swelling in the last affected limb was so much augmented, that the femoral vein was obscured as it had been in the other limb. The pain and tumefaction, however, gradually subsided in both extremities, the general health improved, and on the 12th February no pain whatever was experienced, the lower limbs being merely very weak, and the ankles and legs slightly swelled. No induration or enlargement could be felt in the course of the femoral vessels. The leeches, anodyne fomentations, and cold lotions, constituted the local treatment—purgatives, opiates, and low diet, according to circumstances, the general. The patient was seen at various times by Dr. Sims, Mr. Arnott, and Mr. Prout. From a foot note it appears that Mrs. P. is still under Dr. Lee's care, and that the symptoms general and local make it highly probable that the iliac and femoral veins on both sides, are partially, if not entirely, obstructed.

So much for the foregoing cases which are certainly amongst the most satisfactory of those that have lately been brought forward. The presence of phlebitis in one was established beyond dispute by post-mortem examination, and in the other no reasonable doubt of its existence can be entertained. We also allow that the cases were fair examples of phlegmatia dolens, which is more than we can say of many others which have been published under that head. Here, however, our admissions for the present stop, nor are we by any means compelled by the evidence adduced to alter our expressed opinions on Dr. Davis's theory, respecting the venous origin of the disease. Neither two nor twenty such cases would suffice to establish phlebitis as the cause, the peculiar cause of the disease, especially when analogy furnishes strong arguments against the assumption.

It is rather singular, that after the present paper of Dr. Lee's had been read, he met with three cases which induced him to comprise them in an appendix to the memoir, as well as to modify somewhat his views. In the main essay he observes as follows:—

"From the preceding facts, as well as from the cases to which I have already alluded, we may, I think, safely conclude, that inflammation of the coats of the iliac and femoral veins in puerperal women does unquestionably give rise to all the phenomena of genuine Phlegmasia Dolens. It remains, however, for future observers to determine, if this venous inflammation be the only cause of the disease, or if cases do not occur wherein the other textures are primarily affected. I may observe, that hitherto no instance of Phlegmasia Dolens has been met with where the glandular, lymphatic, or cellular tissues of the limb have been found diseased, without the veins being also in a morbid condition.

"Whether the inflammation of the coats of the veins in this disease be simple adhesive inflammation, or inflammation of a specific kind connected with the puerperal state, and differing not only in degree of intensity, but in its essential nature from phlebitis after venesection, it is difficult to determine. The peculiar character of the symptoms seems strongly to favour the latter opinion, though it cannot be denied that the disease occasionally assumes the form of common phlebitis, fatal cases having occurred, where pus has been found secreted by the internal coats of the iliac veins, and death caused by inflammation and apostematous deposits of matter in the lungs and other remote organs of the body." 145.

The appendix, however, opens thus:—

"Since the preceding paper was communicated to the Society, the three following cases have come under my observation, in which though an inflammatory affection of the veins undoubtedly existed, yet that degree and kind of swelling of the inferior extremity did not take place, which is considered to be characteristic of Phlegmasia Dolens. In all these cases the phlebitis commenced with pain in the situation of the iliac and femoral veins; but in the second, the inflammation, instead of pursuing the course of the femoral vein down the thigh, appeared to have been arrested at the junction of the saphena interna with this vein, and to have proceeded along the trunk and branches of the superficial vessel to the foot. All these affections of the lower extremities which I have witnessed, I am disposed to regard as varieties of the same disease, and to attribute the absence of general swelling of the limbs to the slight degree of inflammation and obstruction which existed in the iliac and femoral veins." 146.

So we see that the fact of the existence of phlebitis without phlegmatia dolens is as we stated, though the Doctor ventures to account for it, by considering the inflammation to have been too slight for the production of that disease. Almost the whole of the cases of phlebitis which we have witnessed have been fatal, and to them Dr. Lee's conjecture must evidently fail to apply. We are arguing not for victory, but truth; and the remarks which we have deemed it our duty to make, have originated only in a belief, whether right or wrong, of the insufficiency of Dr. Davis's hypothesis. We shall be equally pleased whether it ultimately *prove* to be correct or incorrect, as in either case science will be the gainer, and we shall be convinced.

We can only find space for one of the cases contained in the Appendix, but the three are so much alike, that each supplies a tolerable sample of all. We shall take at random the second on the list.

*Case.* Mrs. ——, æt. 37, was delivered on the 14th February, 1829, and on the 21st was attacked with pain in the left inguinal region, which extended during the two next days along the inner surface of the thigh and leg to the ankle. On the 23d, the pain was so acute on the middle of the tibia, about an inch from its inner edge, that she applied some leeches to the spot. The pain was soon succeeded by a diffuse swelling in this situation, and, on the 25th, she complained of great weakness in the whole lower extremity, and pain along the inner surface of the thigh and leg. The trunk and principal branches of the internal saphena were enlarged, indurated, and very painful on pressure, and much swelling obtained in their whole course. The integuments, however, though hotter than on the outer surface of the limb, did not pit upon pressure. All was right in the direction of the femoral vein, and along the outer surface of the limb there was neither swelling, increased heat, nor sensibility. The general disturbance was slight, except when the pain was severe, and then it was of short duration. During gestation she enjoyed good health, but observed the veins of the left lower extremity to swell much more than those of the right. On the 27th, much tenderness was experienced in the course of the femoral vein, which, however, could not be distinctly felt. On the 1st March, the swelling along the saphena, especially in the leg, was increased, and its branches both in the thigh and leg were hard, knotted, and very painful. The integuments along the inner surface of the thigh and leg were hot, but retained their natural colour, and did not pit, except along the front of the tibia.

" March 10th. She suffers much more from pain in the leg, particularly along the exterior surface from the ankle to the knee joint, where several indurated branches of veins can be felt. The whole leg is now affected with a considerable degree of swelling, without increased heat or tension of the integuments. The branches of the internal saphena vein in the thigh are still hard and painful, and the femoral vein is now perceived to be enlarged and indurated, under Poupart's ligament, though but little sensible when pressed. The limb is extremely feeble, and great pain is produced by attempting to extend it." 150.

On the 18th the leg and ankle were œdematous, and the extremity was very weak. The coats of the saphena were still thick and indurated, though perfectly free from pain. The general health was good.

He who can see in the details of the above case much more than a common attack of chronic inflammation of the saphena vein, must be of a very *phlegmatic* habit indeed. We return many thanks to Dr. Lee for the pleasure derived from perusing his interesting paper, which is drawn up with modesty, perspicuity both of thought and language, and good sense.

